# Supplementary material for: Structure-Functional Analysis of Human Cytochrome P450 2C8 Using Directed Evolution
Source: Pharmaceutics. 2021 Sep 9;13(9):1429. doi: 10.3390/pharmaceutics13091429 (PMC8469462; doi:10.3390/pharmaceutics13091429)
Supplement: Supplementary file 1 [file pharmaceutics-13-01429-s001.zip › pharmaceutics-1358718-supplementary.pdf]

pharmaceutics

MDPI

Supplementary Materials: Structure-Functional Analysis of Human Cytochrome P450 2C8 Using Directed Evolution

Rowoon Lee, Vitchan Kim, YoungJin Chun and Donghak Kim

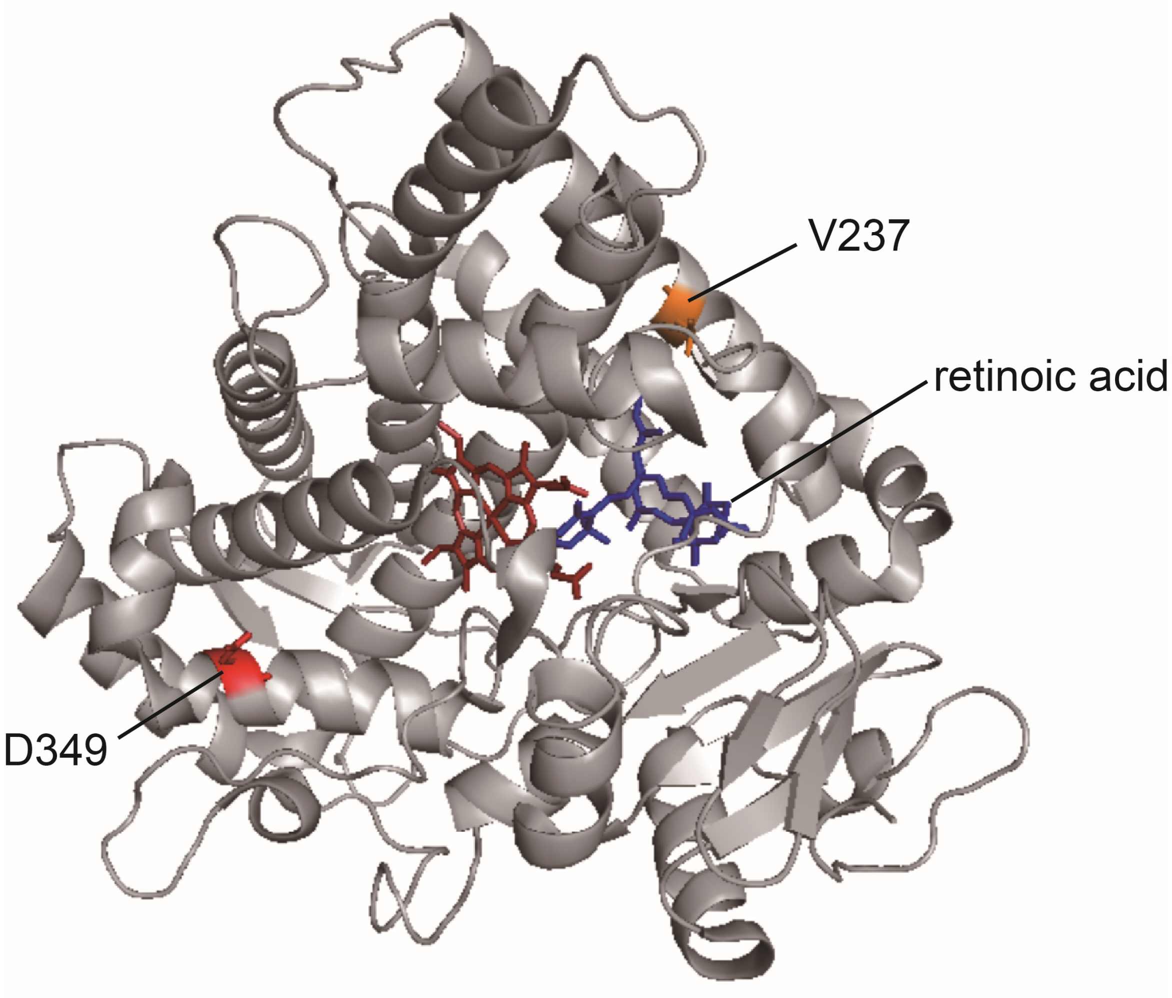

Figure S1. Mutated amino acid residues in the active site of CYP2C8. The positions of the D349Y and V237A mutations are indicated by color in the ribbon diagram of the CYP2C8 X-ray crystal structure (PDB entry code: 2NNH). Heme and retinoic acid molecules are shown in red and navy, respectively.
